# A case report of massive pulmonary tuberculosis and newly diagnosed systemic lupus erythematosus with complications

**DOI:** 10.1002/ccr3.9407

**Published:** 2024-09-04

**Authors:** Marina Vasilj, Tanja Zovko, Kristina Galic, Marija Goluza Sesar, Natasa Pejanovic Skobic, Katica Pavlovic

**Affiliations:** ^1^ Department of Lung Diseases and Tuberculosis University Clinical Hospital Mostar Mostar Bosnia and Herzegovina; ^2^ Faculty of Medicine University of Mostar Mostar Bosnia and Herzegovina; ^3^ Clinic for Neurology University Clinical Hospital Mostar Mostar Bosnia and Herzegovina; ^4^ Clinic for Urology University Clinical Hospital Mostar Mostar Bosnia and Herzegovina

**Keywords:** prolonged cough, pulmonary embolism, pulmonary tuberculosis, systemic lupus erythematosus

## Abstract

The diagnosis of extensive pulmonary tuberculosis, especially in young people, should take into account the possibility of an associated systemic autoimmune disease. Infections remain an important cause of morbidity and mortalityin systemic lupus erythematosus. This case illustrates the importance of recognizing the association of systemic autoimmune diseases and infections and the need for a multidisciplinary approach.

## 1 INTRODUCTION

Although there has been significant improvement in outcome and survival in systemic lupus erythematosus (SLE), infections remain an important cause of morbidity and mortality.^1^ They can appear in the early or late stages of SLE, and account for about 14%–50% of hospitalizations.^2^, ^3^ The increased risk of infections is attributed to disease‐related factors that lead to dysregulation of the immune system and the use of immunosuppressive drugs.^4^ About half of patients with SLE experience a serious infection during the course of the disease, and 11%–23% of hospitalizations among patients with SLE are due to infections.^5^ One‐third of SLE‐related deaths can be attributed to an infectious organism.^6^, ^7^ The World Health Organization (WHO) Global Tuberculosis Report 2022 estimates 10.6 million new tuberculosis (TB) cases in 2021, most common in Southeast Asia (45%), Africa (23%), the Western Pacific (18%), and the Eastern Mediterranean (8.3%).^8^ There was a smaller share in America (2.9%), and Europe (2.2%).^8^ The estimated number of deaths from TB in the world is about 1.6 million, of which about 187,000 were human immunodeficiency virus (HIV) positive patients.^8^ The incidence of TB in patients with SLE is seven times higher than that in healthy populations in developed countries and is estimated at 5% in developing countries.^9^

## 2 CASE HISTORY/EXAMINATION

A 22‐year‐old patient was hospitalized due to febrility, and a non‐productive cough that had lasted for the past 9 months. He also noticed that he had poor effort tolerance, and in the last month had lost 10 kg of body weight. He was not on any drugs in chronic therapy. He did not drink alcohol nor smoke cigaretts. On admission, the patient was conscious, oriented, hypotensive (100/60 mmHg), subfebrile (37.4*C), acyanotic (Sp02: 92%) at room temperature, and of asthenic structure. The skin and visible mucous membranes were extremely pale, without rash. On auscultation breath sound intensity was reduced bilaterally was verified on both sides. Sinus tachycardia was noted in the electrocardiogram (ECG) record upon admission. On admission, laboratory findings showed anemia with elevated inflammatory parameters, and a liver lesion (Table 1).

**TABLE 1:** Laboratory findings.

Investigation	Value	Reference range with unit
WBC	12.2	3.4–9.7 × 10^9^/L
Neutrophyli	10.26	2.06–6.49 × 10^9^/L
Hemoglobin	103	138–175 g/L
ESR	34	2–13 mm/h
Creatinine	32	64–104 μmol/L
Sodium	130	137–146 mmol/L
Chloride	95	97–108 mmol/L
CRP	107.3	0.0–5.0 mg/L
AST	123	11–38 U/L
ALT	45	12–48 U/L
LDH	453	124–241 U/L
GGT	93	23–91 U/L
Albumin	15.6	40.6–51.4 g/L

Abbreviations: ALT, alanine transaminase; AST, aspartate aminotransferase; CRP, C‐reactive protein; ESR, Erythrocyte sedimentation rate; GGT, gamma‐glutamyl transferase; LDH, lactic acid dehydrogenase; WBC, white blood cell.

## 3 METHODS (DIFFERENTIAL DIAGNOSIS, INVESTIGATIONS AND TREATMENT)

**FIGURE 1:**
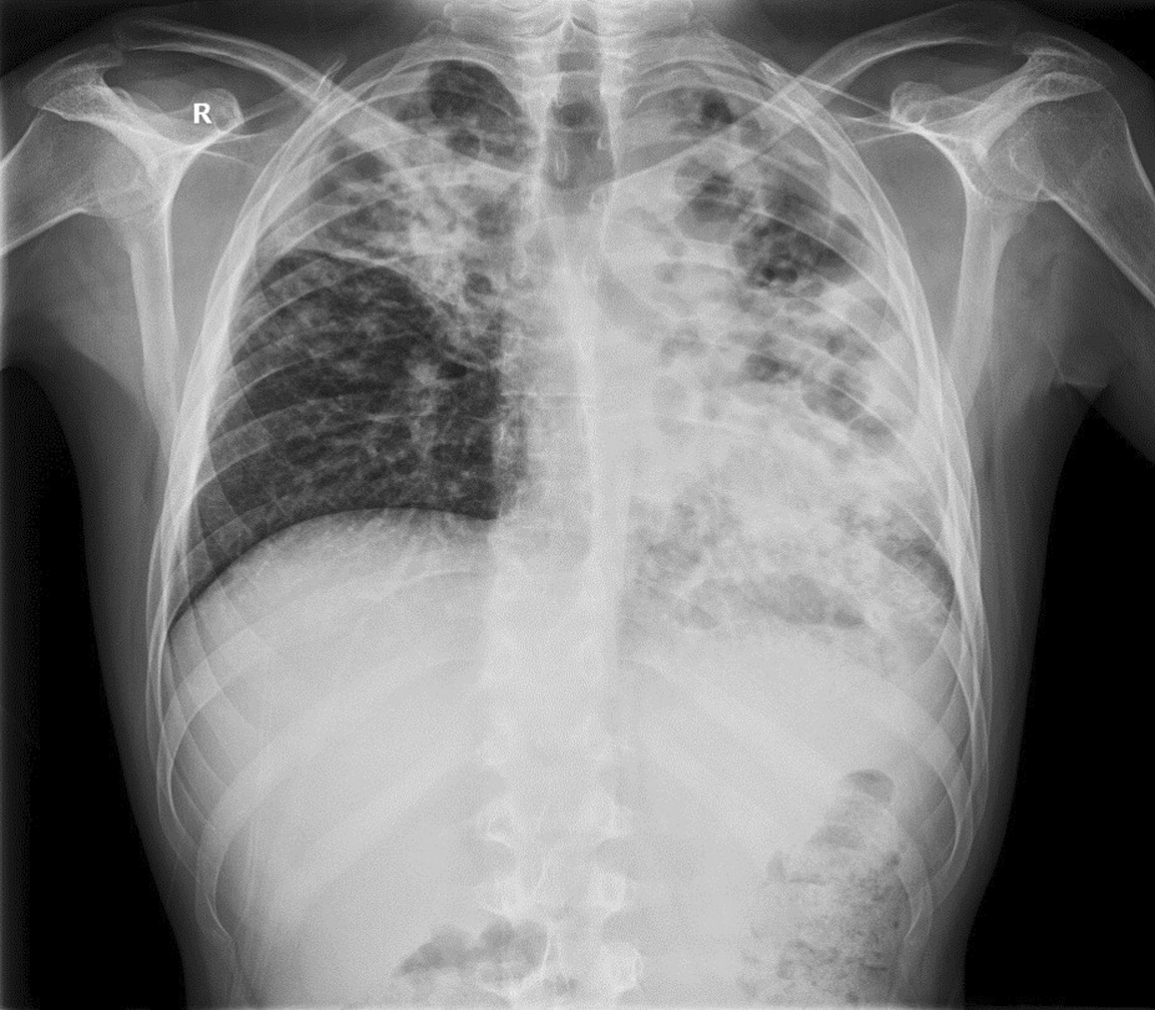
Chest X‐ray on admission.

A radiological image of the lungs was a completely changed. On the left side of the lungs completely changed architecture of the lung parenchyma with irregular, mostly oval transparencies dominantly in the upper and middle lung fields, and an air bronchogram in the lower lung field with spotty shadowing of the lung parenchyma basally is presented. On the right in the upper, partly in the middle lung field, inhomogeneous shadowing of the lung parenchyma with retraction of the horizontal interlobe and right hilus (Figure 1).

**FIGURE 2:**
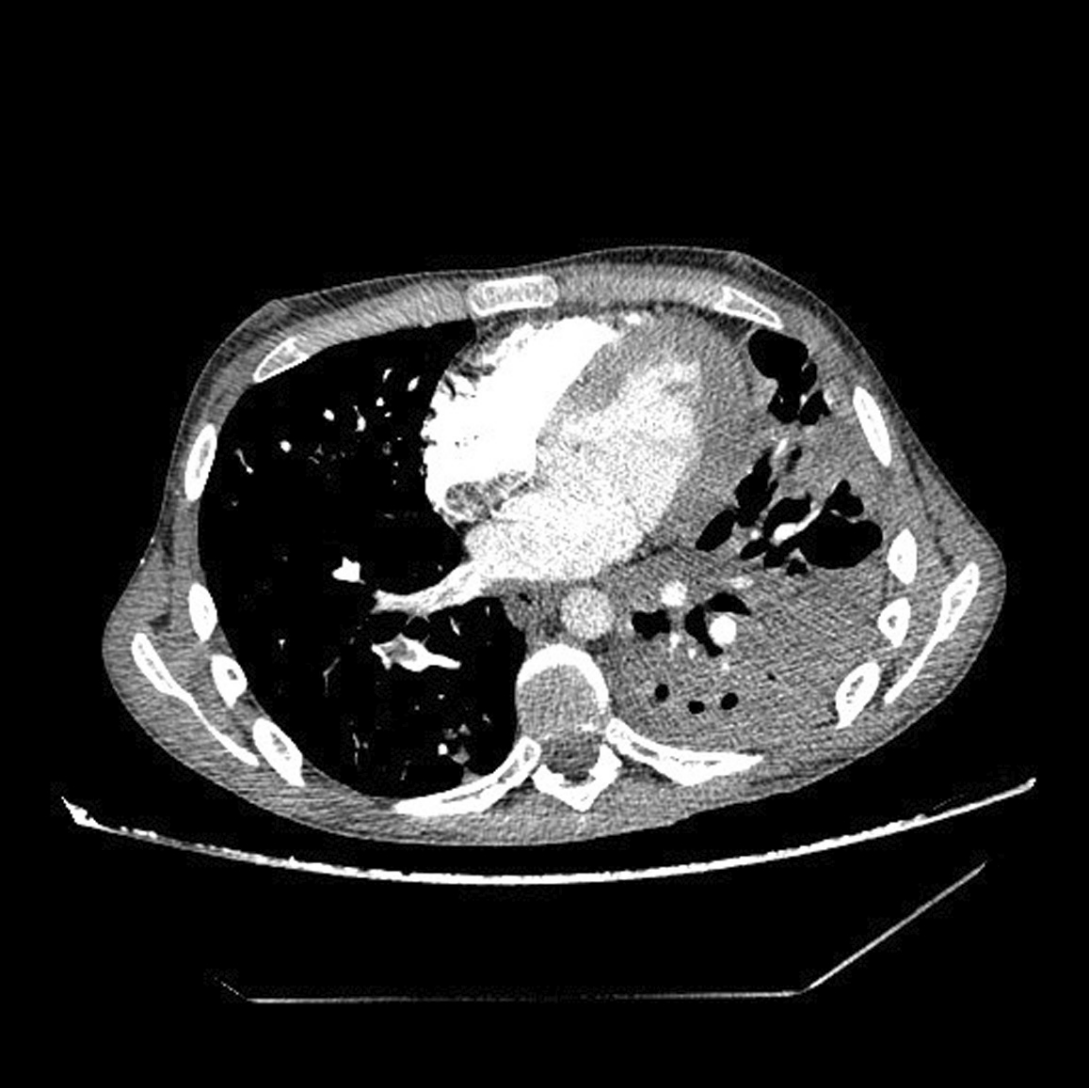
MSCT‐pulmonary angiography of the pulmonary arteries.

**FIGURE 3:**
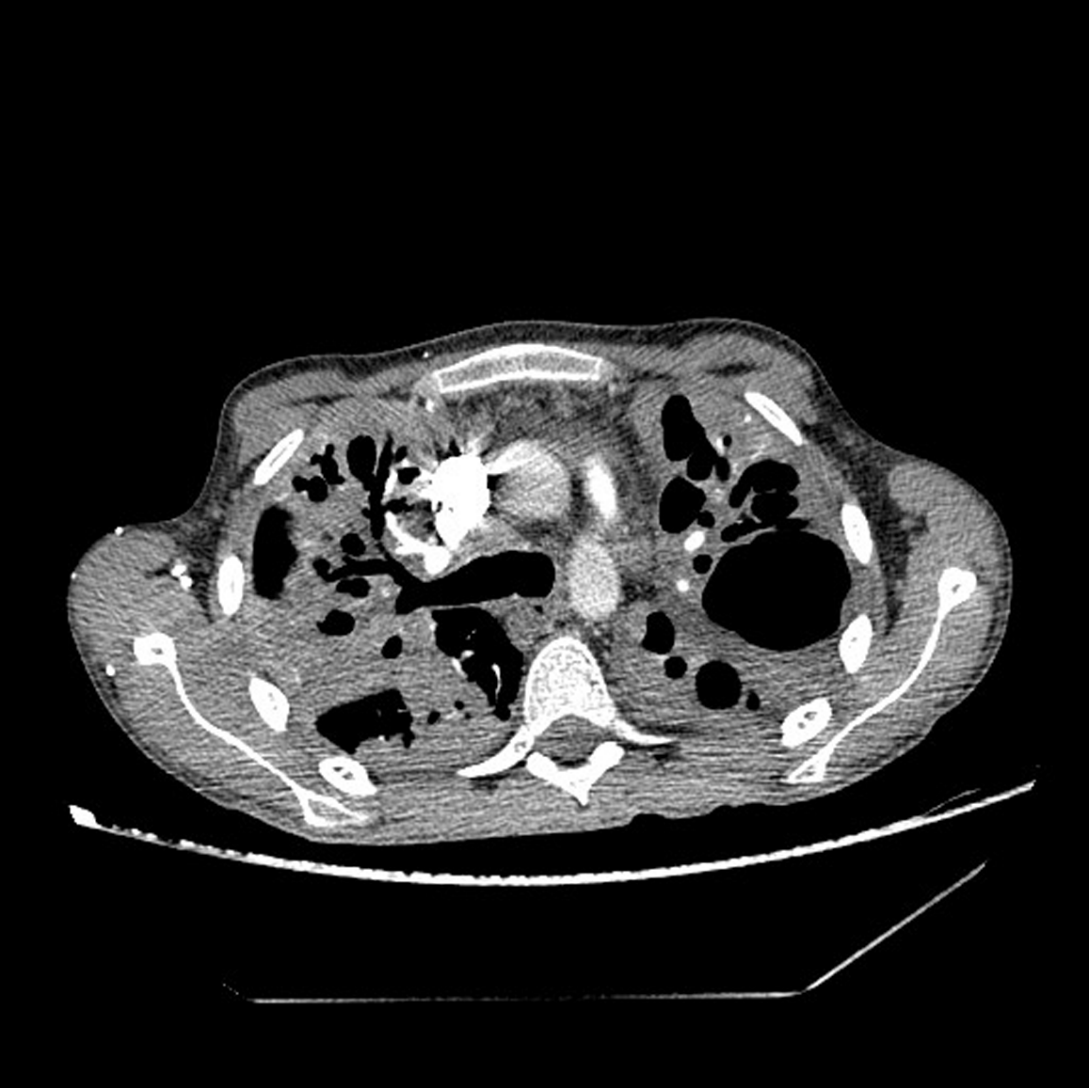
MSCT‐pulmonary angiography of the pulmonary arteries.

Immediately upon admission, sputum was analyzed for *Mycobacterium tuberculosis* (M. tb), which was directly positive (+++ [>100]), after which quadruple anti‐tuberculosis therapy (ATT) (rifampin, isoniazid, pyrazinamide, and ethambutol) in a reduced dose according to body weight (body weight less than 50 kg) was included with gastroprotection, albumin replacement therapy, and preventive anticoagulant therapy with low molecular weight heparin, and continuous oxygen therapy with a flow rate of 4 L/min., and antipyretics. According to the current guidelines due to severe acute respiratory syndrome coronavirus 2 (SARS‐CoV‐2) epidemics, a PCR means polymerase chain reaction (PCR) test for SARS‐CoV‐2 was performed at the reception, which was positive, and the epidemiological measures, and symptomatic treatment was followed. After 14 days of hospitalization, a control test for SARS‐CoV‐2 was performed, which arrived negative. On the 10th day of hospitalization, the patient developed a rash on the skin, for which a dermatologist was consulted. ATT therapy was stopped and drugs were gradually introduced again. Pyrazinamide tablets were excluded from the therapy due to the suspicion that it caused skin allergy. Because of the massive pulmonary infiltrates, streptomycin (750 mg) intramuscularly was added for 2 months. The course of the disease was complicated on the 17th day of treatment when the patient experienced chest pain with recorded sinus tachycardia in the ECG, and due to suspected pulmonary embolisman urgent multislice computed tomography (MSCT) angiography of the pulmonary arteries was performed, which showed “riding” and partial filling defects in the branches of the right pulmonary artery for the lower lung lobe (Figures 2 and 3). After confirming the diagnosis of pulmonary embolism, low molecular weight heparin in full therapeutic dose was included.

Due to fever the infectologist was consulted. Enterococcus spp. was isolated in urine culture. In therapy, according to the antibiogram, intravenous linezolid was included. The control urine culture was negative. Due to the suspicion of an associated immunological diseases because of prolonged febrile state, swelling and joint pain, weight loss, and Raynaud's syndrome an immunologist was also consulted and recommended a complete immunological diagnostic processing. The markers for blood‐transmitted diseases arrived negative (Table 2).The SLICC Criteria for SLE were met for making the diagnosis of lupus.^10^ One clinical criterion (arthritis) and four laboratory criteria were present (positive antibody to double‐stranded deoxyribonucleic acid (antids‐DNA), positive anticardiolipin antibodies, positive lupus anticoagulant (LAC), low C4, positive Coombs test in the absence of hemolytic anemia) (Table 3). The Sapporo criteria for the classification of the antiphospholipid syndrome (APS) were met.^11^

**TABLE 2:** Laboratory of markers for blood transmitted disease findings.

Investigation	Value
HBsAg	Negative (0,03)
Anti HCV	Negative (0,05)
HIV Ag/Ab	Negative (0,326)
Anti HBcIgM	Negative (0,11)
Anti HBs	1.000,00
HBe Ag	Negative (0,01)
Anti HBe	Negative (2.38)
Anti HAV IgM	Negative (0,1)
Anti HAV IgM + IgG	Negative (2,46)
Anti HBc /IgM + IgG	Negative (2,41)

**TABLE 3:** Laboratory of imunology findings.

Investigation	Value	Reference range with unit
C3	1.800	0.90–1.80 g/L
C4	0.090	0.1–0.4 g/L
Rheumatoid factor	32.86	0.00–14.00 IU/mL
ds‐DNA	114.0	>15 positive IU/mL
c‐ANCA (PR‐3)	0.4	>3.0 IU/mL
p‐ANCA (MPO)	0.2	>5 IU/mL
Anti‐GBM	>1.0	>10 IU/mL
ENA	0.1	>1.0 ratio
Anti‐CCP	3.1	>10 IU/mL
LAC	1.43	<3.0 mmol/L
Antibodies against b2 glycoprotein 1‐IgG	4.1	>10 IU/mL
Antibodies against b2 glycoprotein 1‐IgM	1.6	>10 IU/mL
Antibodies against cardiolipin‐IgG	9.5	>40 GPL‐IU/mL
Antibodies against cardiolipin‐IgM	18.0	>40 MPL‐IU/mL
Coombs indirect	Negative	
Coombs direct	Positive	

**FIGURE 4:**
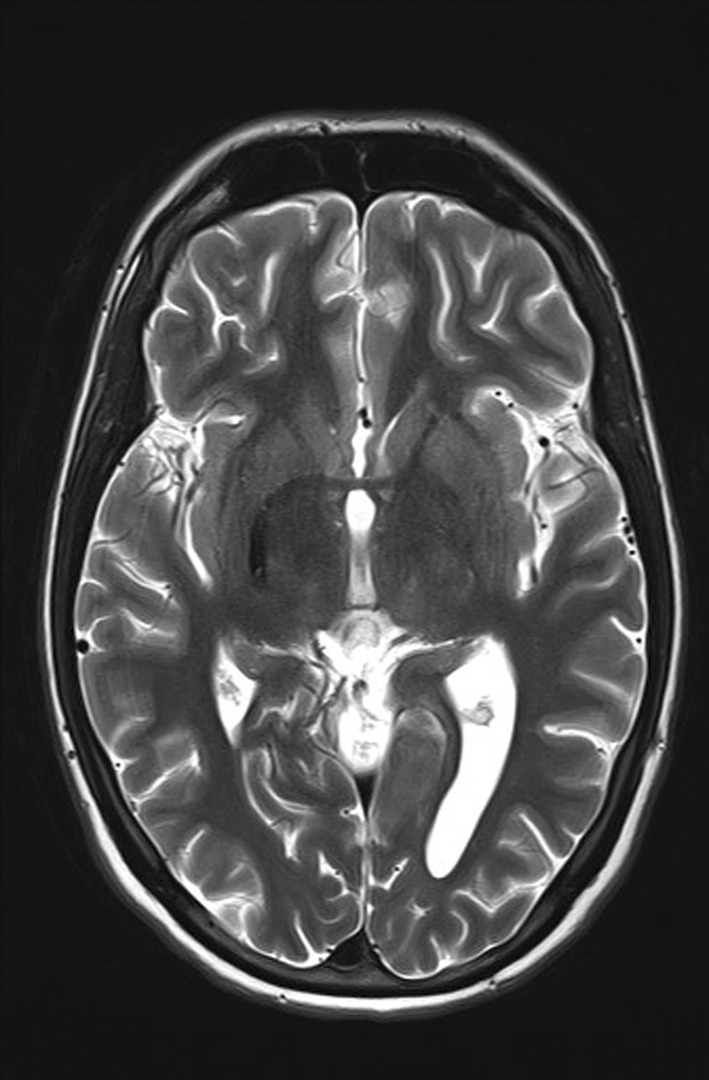
Magnetic resonance imaging (MRI) and magnetic resonance angiogram (MRA) of the brain.

**FIGURE 5:**
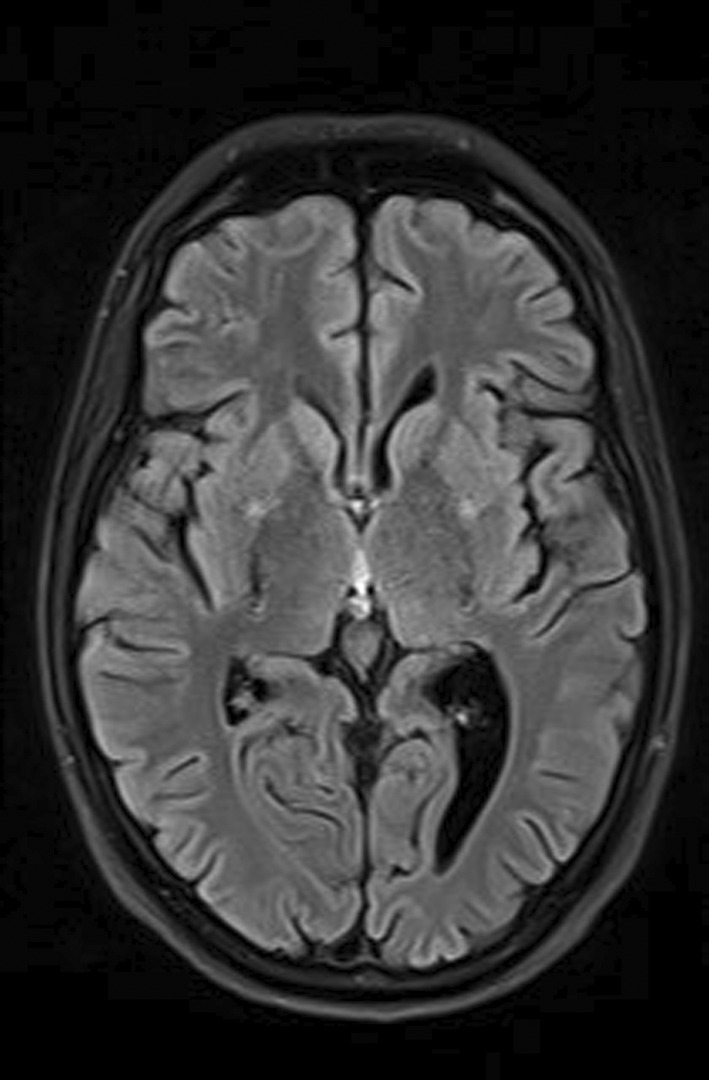
Magnetic resonance imaging (MRI) and magnetic resonance angiogram (MRA) of the brain.

**FIGURE 6:**
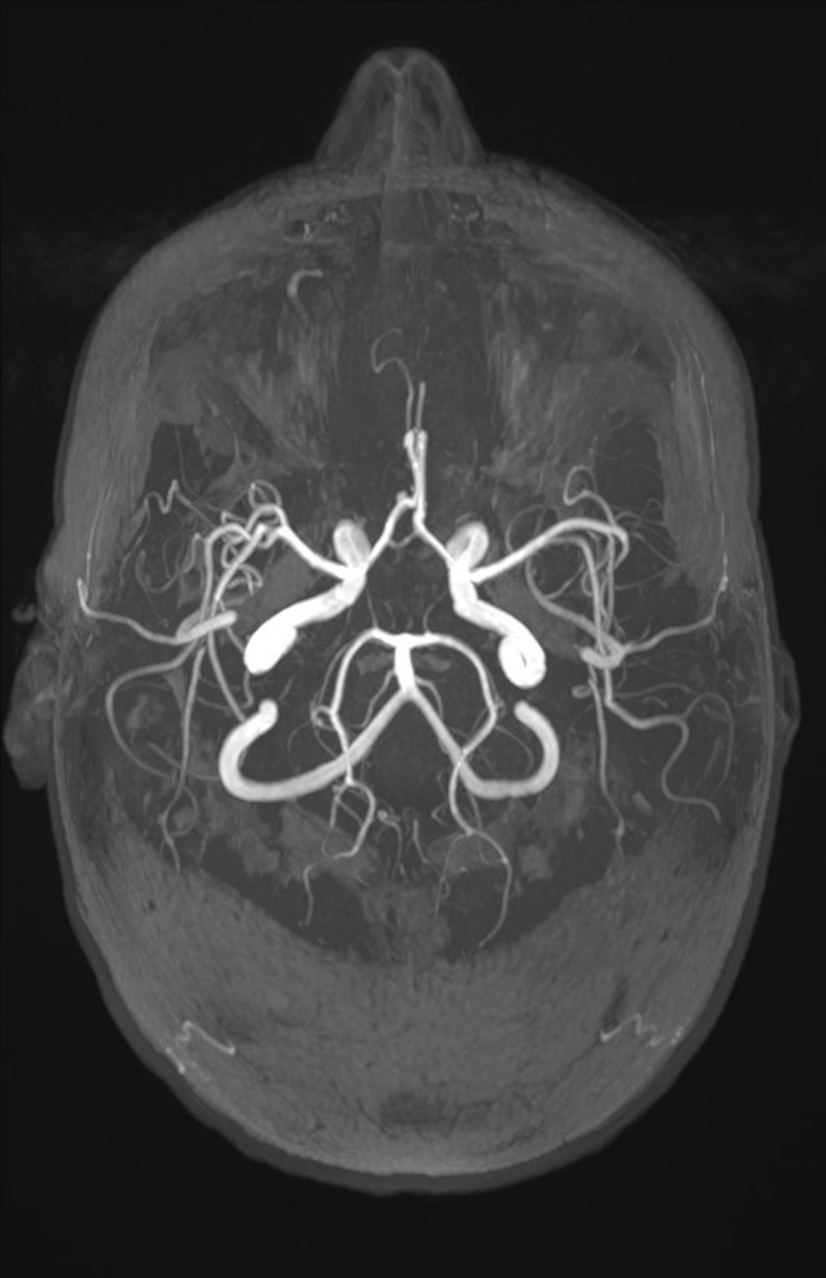
Magnetic resonance angiogram (MRA) using 3 D TOF techniques.

**FIGURE 7:**
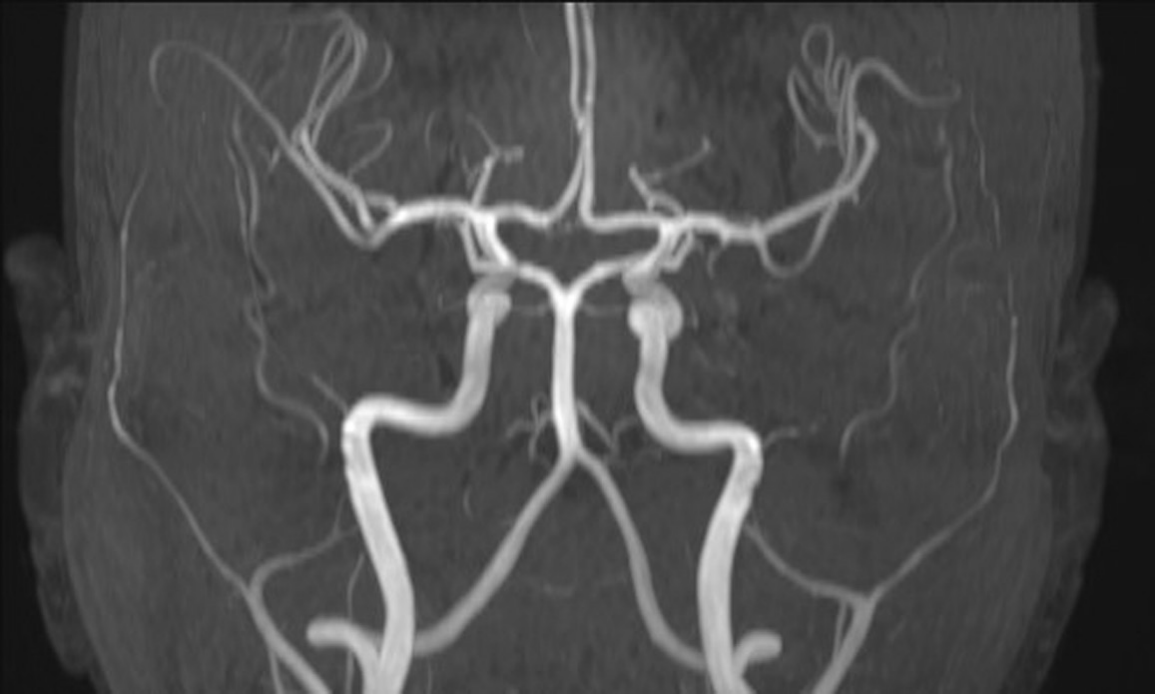
Magnetic resonance angiogram (MRA) using 3 D TOF techniques.

As part of the diagnostic recommendation by the immunologist, magnetic resonance imaging (MRI), and magnetic resonance angiogram (MRA) of the brain were also performed. The finding was unremarkable (Figures 4 and 5). In MRA using 3 DTOF techniques. The finding was unremarkable with an adequate course without clearly observed signs of aneurysm or other arteriovenous malformation (AVM) (Figures 6 and 7).

## 4 CONCLUSION AND RESULTS (OUTCOME AND FOLLOW‐UP)

**FIGURE 8:**
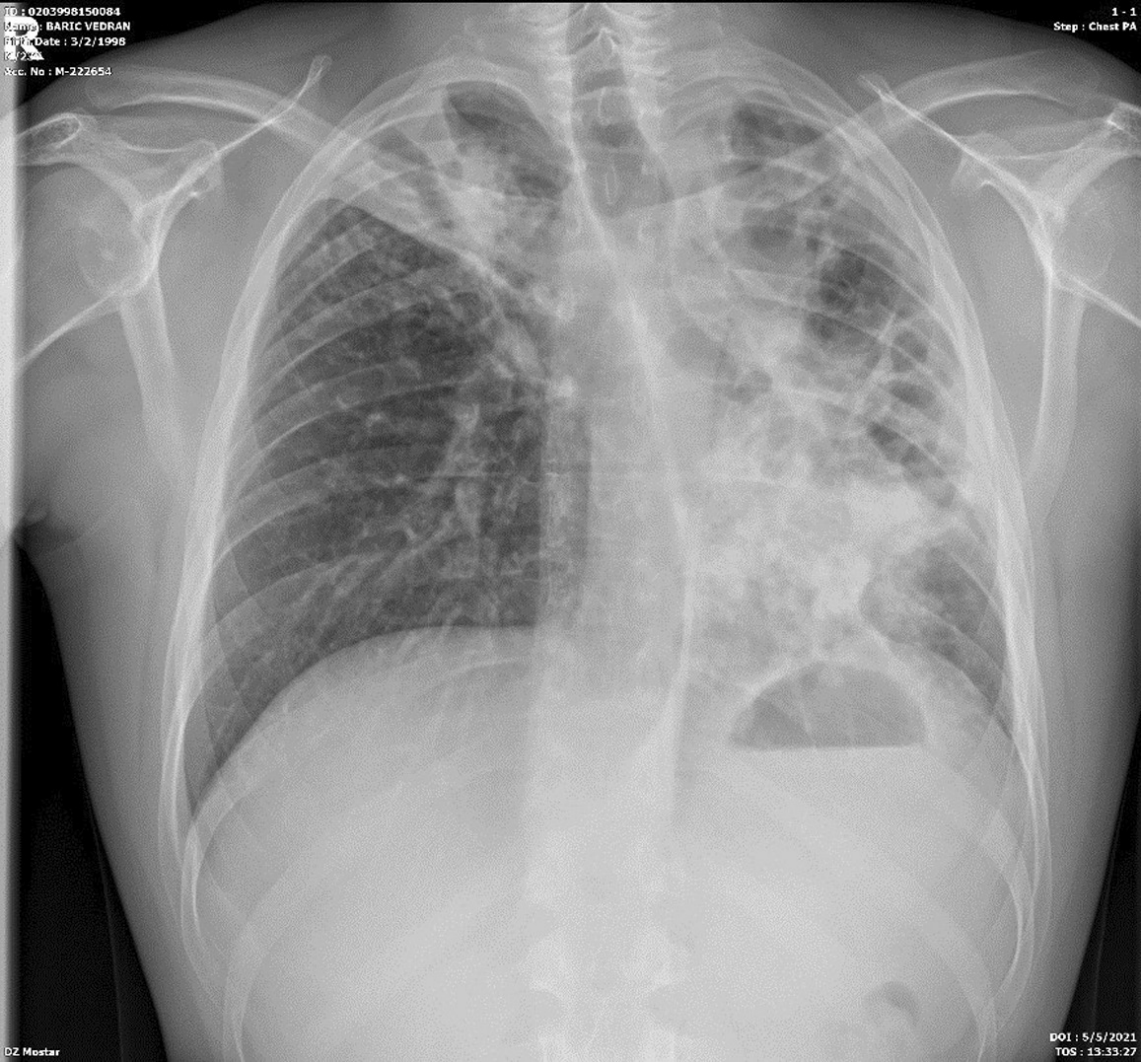
Chest X‐ray on discharge showed improvement.

Based on the above, therapy for SLE was prescribed along with ATT therapy, which led to subjective and clinical improvement and a febrile state. The therapy for SLE was administered (decortine, azathioprine, and hydroxychloroquine).The patient gained 10 kg in body weight during his stay in the hospital. After 110 days of hospitalization, with the control sputum for *M. Tuberculosis* being negative, after the control x‐ray of the lungs (Figure 8), showed improvement, the patient was discharged from the hospital. ATT therapy was conducted for 6 months (two months of the initiation phase, and the continuation phase for 4 months). Regular controls by a pulmonologist and immunologist were performed.

## 5 DISCUSSION

There is a complex interaction between SLE and TB.^12^, ^13^ Patients with SLE have an increased risk of developing infections, including TB due to their impaired immune defence and use of immunosuppressive agents. In patients with SLE, TB is more likely to be extrapulmonary. Involvement is more severe, and relapses occur more often.^11^ Infections can also trigger autoimmune diseases and contribute to the induction and exacerbation of SLE.^12^, ^14^ Diagnosing TB in patients with SLE is often a challenging task because they have many similar manifestations including fever, weight loss, and constitutional disturbances. In addition, pulmonary, and neurological manifestations can occur in both SLE and TB.^15^ Therefore, common rheumatic diseases can be misdiagnosed as general infections, leading to a very high rate of misdiagnosis and underdiagnosis.

Many exposed persons in endemic areas will be at risk of TB reactivation with autoimmune diseases or immunosuppressive drugs, so a multidisciplinary approach is needed to identify patients at increased risk of TB who will benefit from prophylactic therapy.

## AUTHOR CONTRIBUTIONS

**Marina Vasilj:** Conceptualization; investigation; methodology; project administration; supervision; visualization; writing – original draft. **Tanja Zovko:** Conceptualization; data curation; investigation; visualization; writing – original draft. **Kristina Galic:** Methodology; supervision; validation; visualization. **Marija Goluza Sesar:** Funding acquisition; methodology; resources. **Natasa Pejanovic Skobic:** Formal analysis; resources; software. **Katica Pavlovic:** Data curation; methodology; validation.

## FUNDING INFORMATION

This research received no specific grants from any funding agency in the public, commercial, or not‐for‐profit sector.

## CONFLICT OF INTEREST STATEMENT

The authors declare no relationships, including financial or professional, which may pose a competing interest.

## CONSENT

Written informed consent was obtained from the patient to publish this report in accordance with the journal's patient consent policy.

## Data Availability

The data that support the findings of this study are available from the corresponding author upon reasonable request.

## References

[1] Moreno‐Torres V , Martínez‐Urbistondo M , Gutiérrez‐Rojas A , et al. Impact of severe infections in SLE: an observational study from the Spanish national registry. Lupus Sci Med. 2022;9:e000711. doi:10.1136/lupus-2022-000711 36283745 PMC9608526

[2] Barber C , Gold WL , Fortin PR . Infections in patients with lupus: perspectives on prevention. Curr Opin Rheumatol. 2011;23:358‐365. doi:10.1097/BOR.0b013e3283476cd8 21532484

[3] Fessler BJ . Infectious diseases in systemic lupus erythematosus: risk factors, treatment and prophylaxis. Best Pract Res Clin Rheumatol. 2002;16:281‐291. doi:10.1053/berh.2001.0226 12041954

[4] James JA , Sestak AL , Vista ES . SLE and infection. In: Wallace DJ , Hahn BH , eds. DUBOIS' Lupus Erythematosus and Related Syndromes. ELSEVIER Saunders; 2013:555‐562.

[5] Petri M , Genovese M . Incidence of and risk factors for hospitalizations in systemic lupus erythematosus: a prospective study of the Hopkins lupus cohort. J Rheumatol. 1992;19:1559‐1565. 1464868

[6] Edwards CJ , Lian TY , Badsha H , Teh CL , Arden N , Chng HH . Hospitalization of individuals with systemic lupus erythematosus: characteristics and predictors of outcome. Lupus. 2003;12:672‐676. 14514129 10.1191/0961203303lu452oa

[7] Hellmann DB , Petri M , Whiting‐O'Keefe Q . Fatal infections in systemic lupus erythematosus: the role of opportunistic organisms. Medicine (Baltimore). 1987;66:341‐348. 3626846 10.1097/00005792-198709000-00002

[8] World Health Organization . Global Tuberculosis Report 2022. World Health Organization; 2022 License: CC BY‐NC‐SA 3.0 IGO.

[9] Singh JA , Hossain A , Kotb A , Wells G . Risk of serious infections with immunosuppressive drugs and glucocorticoids for lupus nephritis: a systematic review and network meta‐analysis. BMC Med. 2016;14:137. doi:10.1186/s12916-016-0673-8 27623861 PMC5022202

[10] Kostopoulou M , Mukhtyar CB , Bertsias G , Boumpas DT , Fanouriakis A . Management of systemic lupus erythematosus: a systematic literature review informing the 2023 update of the EULAR recommendations. Ann Rheum Dis. 2024:ard‐2023‐225319. doi:10.1136/ard-2023-225319 PMC11503129 38777375

[11] Al‐Banaa K , Herrman E , Antonios B , et al. Assessing the rate of compliance with the revised Sapporo/Sydney criteria in diagnosing antiphospholipid syndrome: a multicenter retrospective analysis. Blood. 2022;140:2795‐2796. doi:10.1182/blood-2022-157217

[12] Prabu V , Agrawal S . Systemic lupus erythematosus and tuberculosis: a review of the complex interactions of complicated diseases. J Postgrad Med. 2010;56:244‐250. doi:10.4103/0022-3859.68653 20739781

[13] Balbi GGM , Machado‐Ribeiro F , Marques CDL , Signorelli F , Levy RA . Interaction between tuberculosis and systemic lupus erythematosus. Curr Opin Rheumatol. 2018;30:395‐402. doi:10.1097/BOR.0000000000000493 29438163

[14] Ribeiro FM , Szyper‐Kravitz M , Klumb EM , et al. Can lupus flares be associated with tuberculosis infection? Clin Rev Allergy Immunol. 2010;38:163‐168. doi:10.1007/s12016-009-8149-7 19548122

[15] Goletti D , Delogu G , Matteelli A , Migliori GB . The role of IGRA in diagnosing tuberculosis infections, distinguishing them from active tuberculosis, and making a decision on the initiation of treatment or preventive therapy of tuberculosis infections. Int J Infect Dis. 2022;124:S12‐S19. doi:10.1016/j.ijid.2022.02.047 35257904

